# How variable progenitor clones construct a largely invariant neocortex

**DOI:** 10.1093/nsr/nwad247

**Published:** 2023-09-15

**Authors:** Zhongfu Shen, Jiajun Yang, Qiangqiang Zhang, Kuiyu Wang, Xiaohui Lv, Xiaolin Hu, Jian Ma, Song-Hai Shi

**Affiliations:** New Cornerstone Science Laboratory, IDG/McGovern Institute for Brain Research, Tsinghua-Peking Center for Life Sciences, Beijing Frontier Research Center for Biological Structure, School of Life Sciences, Tsinghua University, Beijing 100084, China; New Cornerstone Science Laboratory, IDG/McGovern Institute for Brain Research, Tsinghua-Peking Center for Life Sciences, Beijing Frontier Research Center for Biological Structure, School of Life Sciences, Tsinghua University, Beijing 100084, China; New Cornerstone Science Laboratory, IDG/McGovern Institute for Brain Research, Tsinghua-Peking Center for Life Sciences, Beijing Frontier Research Center for Biological Structure, School of Life Sciences, Tsinghua University, Beijing 100084, China; Department of Computer Sciences, Tsinghua University, Beijing 100084, China; New Cornerstone Science Laboratory, IDG/McGovern Institute for Brain Research, Tsinghua-Peking Center for Life Sciences, Beijing Frontier Research Center for Biological Structure, School of Life Sciences, Tsinghua University, Beijing 100084, China; College of Life Sciences, Nankai University, Tianjin 300071, China; Department of Computer Sciences, Tsinghua University, Beijing 100084, China; New Cornerstone Science Laboratory, IDG/McGovern Institute for Brain Research, Tsinghua-Peking Center for Life Sciences, Beijing Frontier Research Center for Biological Structure, School of Life Sciences, Tsinghua University, Beijing 100084, China; New Cornerstone Science Laboratory, IDG/McGovern Institute for Brain Research, Tsinghua-Peking Center for Life Sciences, Beijing Frontier Research Center for Biological Structure, School of Life Sciences, Tsinghua University, Beijing 100084, China; Chinese Institute for Brain Research, Beijing 102206, China

**Keywords:** neocortex, radial glial progenitor, intermediate progenitor, neurogenesis, clonal variability, layer formation

## Abstract

The neocortex contains a vast collection of diverse neurons organized into distinct layers. While nearly all neocortical neurons are generated by radial glial progenitors (RGPs), it remains largely unclear how a complex yet organized neocortex is constructed reliably and robustly. Here, we show that the division behavior and neuronal output of RGPs are highly constrained with patterned variabilities to support the reliable and robust construction of the mouse neocortex. The neurogenic process of RGPs can be well-approximated by a consistent Poisson-like process unfolding over time, producing deep to superficial layer neurons progressively. The exact neuronal outputs regarding layer occupation are variable; yet, this variability is constrained systematically to support all layer formation, largely reflecting the variable intermediate progenitor generation and RGP neurogenic entry and exit timing differences. Together, these results define the fundamental features of neocortical neurogenesis with a balanced reliability and variability for the construction of the complex neocortex.

## Teaser

This study conducted a systematic quantitative analysis of the number and laminar distribution of the neuronal output by individual progenitors, revealing an intricate balance between reliability and variability for the effective construction of the complex neocortex.

## INTRODUCTION

The neocortex consists of millions to billions of highly diverse neurons that are organized into distinct layers to support its function. From the pial surface to the inner white matter, there are typically six layers (L1-6) of neurons. Each layer harbors specific types of neurons with characteristic gene expression, morphology, and biophysical and functional properties [1–5]. In particular, the superficial layers (L2-3) largely contain neurons projecting within the neocortex either ipsilaterally or contralaterally, whereas the deep layers (L5-6) mainly possess neurons projecting out of the neocortex, such as to the thalamus, the hindbrain, or the spinal cord [6,7]. Layer 4 in the sensory areas contains neurons that directly receive sensory inputs from the thalamus. The laminated organization of neurons is essential to circuitry assembly and neocortical function. Abnormal production of neurons or malformation of the laminar structure often leads to neocortical dysfunction and severe neurological diseases, such as epilepsy, autism spectrum disorder, and intellectual disabilities [8–11]. A fundamental and largely unresolved question concerns how the complex yet organized neocortex is constructed reliably and effectively during development.

The laminated neocortex emerges progressively over time and is tied to the orderly generation and migration of newborn neurons [12–14]. Neurons are generated by dividing neural progenitor cells in the ventricular zone (VZ) near the lateral ventricle and the adjacent subventricular zone (SVZ), and migrate radially to constitute the future neocortex [15,16]. Radial glial cells are the predominant neural progenitor cells in the developing neocortex. Originating from the neuroepithelial cells, radial glial progenitors (RGPs) reside in the VZ with a characteristic bipolar morphology, possessing a long basal radial glial fiber extending to the pial surface and a short apical endfoot reaching the lateral ventricle [17–19]. During neocortical development, RGPs actively divide at the VZ surface. Initially (e.g. prior to embryonic day, E, 11–12 in mice), RGPs largely divide symmetrically to amplify the progenitor cell pool [20–22]. Subsequently, they undergo asymmetric division to produce neurons or transit amplifying progenitors that divide in the SVZ to produce neurons and, at the same time, to self-renew. The typical transit amplifying progenitors include intermediate progenitors (IPs) with a limited division potential (e.g. largely one round of division in mice) and outer SVZ radial glial progenitors (oRGs; also called basal RGPs, bRGs, or transit RGPs, tRGs) with more division potentials that are more abundant in higher-order species, such as the primates [23,24]. Notably, the pseudostratified organization in conjunction with the unique centrosome positioning dictates that RGPs undergo interkinetic nuclear oscillation coupled with cell cycle progression and divide only at the VZ surface, leading to the wave-like fashion of neurogenesis [25]. Neurons born at a similar time migrate as a cohort along the basal radial glial fibers of their mother RGPs and occupy a similar layer. The late-born neurons migrate and pass the early-born neurons to situate in a more superficial position, resulting in a birthdate-dependent inside-out laminar formation. While the majority of RGPs exit the cell cycle and are depleted at the completion of neurogenesis, a fraction of them continue to divide to produce glial cells, including astrocytes and oligodendrocytes, and contribute to adult neural stem cell formation [26–29].

The organized division behavior of RGPs and the orderly generation and migration of newborn neurons drive the assembly of the complex neocortex. RGPs not only act as the predominant progenitors to divide in order to produce diverse neurons and glial cells, but also guide the radial migration of newborn neurons to reach their final destinations [30,31]. Therefore, RGPs play instrumental roles in controlling neocortex formation. Unraveling the division behavior and neuronal output of RGPs, especially at the single-cell resolution in a quantitative manner, is essential to understanding the development of the complex neocortex. Previous studies have shown that individual RGPs in the developing mouse neocortex undergo an organized program of lineage progression and produce diverse neurons in a unitary fashion [22,32–34]. Notwithstanding, the exact neuronal outputs of individual RGPs, in particular with regard to neuronal laminar identity, appear to be variable. Thus, it remains largely unclear how variable neuronal outputs by individual RGPs reliably and effectively lead to the formation of a largely invariant and complex neocortex.

In this study, we set out to address this fundamental question, in particular, by focusing on delineating the nature and origin of clonal neuronal output variabilities and the basic neurogenesis features underlying the reliable and robust construction of the complex yet largely invariant neocortex. By performing the systematic and quantitative clonal analyses of individual RGPs across the entire phase of neurogenesis in the developing mouse neocortex, we found that the reliable and robust assembly of the complex neocortex largely depends on two basic features of neurogenesis, the stable consecutive RGP asymmetric division framework, which supports reliable and steady neurogenesis, and the variable but constrained IP generation, which permits the variability and robustness for effective formation of all layers.

## RESULTS

### Steady Poisson-like process of neuronal production by individual RGPs

Neocortical neurogenesis in the developing mouse brain largely occurs between E12 and E16 via RGP asymmetric division. To understand the construction of the neocortex, it is essential to delineate the precise behavior and neuronal output of individual RGPs *in vivo* at single-cell resolution. To achieve this, we took advantage of the powerful Mosaic Analysis with Double Markers (MADM) approach and applied it to the developing mouse neocortex [35,36]. In MADM, Cre recombinase-mediated inter-chromosomal recombination in the G_2_ phase of a dividing stem or progenitor cell, reconstitutes the fluorescent markers enhanced green fluorescent protein (EGFP) and tandem dimer Tomato (tdTomato), which are then segregated into the two daughter cells, respectively, following X-segregation (i.e. G_2_-X recombination and segregation) (Fig. S1A in the supplementary materials online). This results in a permanent and distinct (green versus red fluorescence) labeling of two daughter cells as well as their descendents, thereby allowing a direct assessment of the division pattern and total neuronal output of the originally labeled dividing stem or progenitor cell. In addition, upon G_2_-Z recombination and segregation events, or G_1_ or G_0_ recombination events, EGFP and tdTomato are segregated or restored simultaneously in the same cell, resulting in double-labeled (green and red fluorescence) cells.

To assess the neuronal output of individual RGPs during neocortical neurogenesis, we integrated the *Emx1-CreER^T2^* transgene [37], in which the expression of tamoxifen (TM)-inducible Cre recombinase is selectively driven by the *Emx1* promoter in the dorsal telencephalic RGPs that produce neocortical excitatory neurons, with the *MADM-11* system [38] (Fig. S1B). A single dose of TM was administered to timed pregnant females at E12, E13, E14, E15 or E16 to label individual dividing RGPs and consequently their neural progenies (i.e. clones) across the neurogenic period. As expected, discrete G_2_-X clonal clusters of green and red fluorescent neurons with characteristic excitatory neuronal morphology were readily found in the postnatal neocortex (arrowheads), e.g. at postnatal day 21–40 (P21–P40), when neurogenesis and neuronal migration are complete (Fig. S1C). Some clonal clusters also contained glial cells (arrows, Fig. S1C). We then performed a systematic and quantitative analysis of clonal neuronal output of individual RGPs by serial sectioning and three-dimensional (3D) reconstruction of the neocortex to recover all fluorescently labeled neurons (Figs S1C, right and 1A).

RGPs in the developing mouse neocortex transition from symmetric proliferative division to asymmetric neurogenic division at E11–E12 [27,39]. Indeed, we observed symmetrically dividing, proliferative RGP clones with a large cohort of green and red fluorescent neurons occupying both deep and superficial layers labeled at E12 (Fig. S1C, top). These green and red fluorescent neurons represent the progenies of the two daughter RGPs of the originally labeled, symmetrically dividing RGP, respectively. In addition, we observed asymmetrically dividing, neurogenic RGP clones with a minority of neurons in one color located in the deep layer and a majority of neurons in the other color spanning both deep and superficial layers (Fig. S1C, bottom). The minority color labeled neurons represents the progenies of a neuron or an IP, whereas the majority color labeled neurons represents the progenies of a daughter RGP of the originally labeled, asymmetrically dividing RGP.

**Figure 1.**
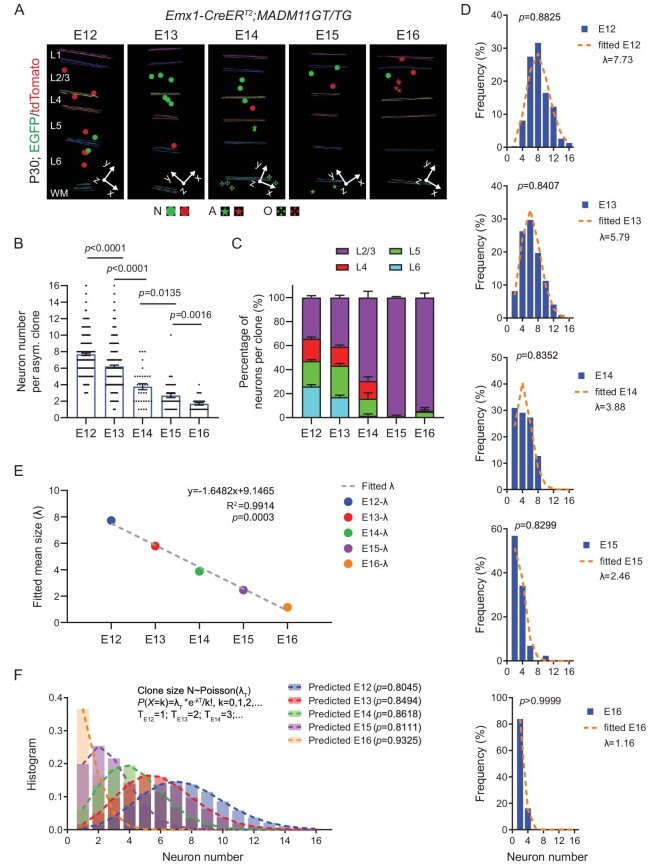
Progressive Poisson-like process of neurogenesis by individual RGPs at different embryonic stages. (A) 3D reconstruction images of the representative clones labeled at different embryonic stages (E12–E16). Colored lines indicate the layer boundary. Round dots indicate the cell bodies of labeled neurons. Stars indicate the cell bodies of labeled astrocytes, and crosses indicate the cell bodies of labeled oligodendrocytes. The x/y/z axes indicate the spatial orientation of the clone with the y axis parallel to the midline and pointing dorsally. Similar display is used in subsequent 3D reconstruction images. L, layer; WM, white matter; N, neuron; A, astrocyte; O, oligodendrocyte. (B) Quantifications of the number of neurons in individual G_2_-X clones labeled at different embryonic stages (E12, *n* = 311; E13, *n* = 320; E14, *n* = 55; E15, *n* = 44; E16, *n* = 31). Bar plots and lines represent mean ± SEM and dots represent individual clones. Statistical analysis was performed using two-sided Mann–Whitney–Wilcoxon test. (C) Percentages of neurons per clones in different layers labeled at different embryonic stages (E12, *n* = 167; E13, *n* = 131; E14, *n* = 33; E15, *n* = 44; E16, *n* = 31). Note a progressive shift in layer output from deep to superficial layers as development proceeds. (D) Histograms of the neuronal number of individual RGP clones labeled at different embryonic stages (E12–E16, blue bars). Each histogram was fitted with a Poisson distribution (orange broken lines). Statistical analysis was performed using Chi-square test between the original histogram and the corresponding Poisson distribution. λ represents the mean number of the Poisson distribution. (E) Linear regression of the mean clone sizes (λ values) for the Poisson fitting at each embryonic stage (E12–E16). The fitted equation with R-square and *p* value is presented as the inset. (F) Size distributions of the predicted clone size at each embryonic stage (E12–E16) by a progressive common Poisson-like process. Statistical analysis was performed using Chi-square test between the original histogram and the corresponding prediction of a common Poisson distribution.

We particularly focused on the asymmetric G_2_-X clones, as they faithfully reflect the complete neurogenic output of individual neurogenic RGPs (Fig. 1A). At the late stage of neurogenesis (e.g. E14–E16), to reliably assess the neurogenic output of RGPs, we examined G_2_-X clones with glial cells that represent RGP, but not IP, clones, as IPs do not generate glial cells [40]. We found that, as development proceeded, the number of neurons in individual neurogenic RGP clones progressively decreased (Fig. 1A, B). Moreover, the neuronal output gradually shifted from deep to superficial layers (Fig. 1A, C). Together, these results suggest that individual RGPs undergo consecutive asymmetric divisions to produce neurons progressively occupying deep and superficial layers, as shown previously [27,41,42].

To quantitatively assess the dynamics of the neurogenic process, we next examined the histogram of clonal neuronal numbers labeled at different developmental stages across all neocortical areas, including the sensory, motor, and prefrontal areas. Remarkably, the clonal neuronal number histogram could be well-approximated by a Poisson-like distribution at each developmental stage across the neurogenic phase (Fig. 1D). More strikingly, the mean number of neurons for the clones labeled at E12–E16 exhibited a linear decrease as development proceeded (Fig. 1E). These results suggest that the entire neurogenic process of RGPs can be described by a common Poisson-like process unfolding over time, leading to a steady rate of neurogenesis. Indeed, the entire neurogenesis process between E12 and E16 with regard to the number of neuronal outputs by individual RGPs could be well-fitted by a progressive Poisson-like process (Fig. 1F). In this process, the average total neuronal output potential of individual RGPs is ∼9 neurons, which is generated at a relatively consistent rate of ∼1.6 neurons per day. Notably, the cell cycle duration of RGPs has been shown to prolong as development proceeds [43]. While we did observe a slightly higher neuronal output rate at the early embryonic stage, the overall neuronal output rate is largely consistent across the entire neurogenic period (Fig. 1E). This is consistent with the notion that the prolongation of cell cycle duration by several hours per day relative to the total cell cycle duration would not drastically affect the neuronal output rate of RGPs. In addition, the progressive cell cycle exit of RGPs along with the development also offset the size of the clone labeled at the early developmental stages [44] (Fig. S2). Taken together, these results suggest that the neurogenesis process in the developing neocortex occurs in a steady and reliable manner, in which RGPs go through multiple rounds of asymmetric division to produce ∼9 neurons in total at a relatively constant rate of ∼1.6 neurons per day across the entire neurogenic phase.

### Variable but constrained neuronal layer output by RGPs

We next examined the clonal neuronal layer output, which exhibits clear variabilities even for the clones labeled at the same developmental stage (Fig. S3). We focused on the asymmetric neurogenic clones labeled at E12 that reflect the more complete neuronal output of individual RGPs. Notably, we found that the neuronal outputs to the deep (5–6) and superficial (2–4) layers within individual clones were often uneven (Fig. S3A). Some clones contained more neurons in the deep layers (Fig. S3A, left), whereas other clones possessed more neurons in the superficial layers (Fig. S3A, right). We quantitatively compared the relative number of neurons in individual clones located in the deep versus superficial layers, and observed a significant anti-correlation (Fig. S3B), indicating that the neuronal layer output by individual RGPs is constrained and balanced. Should an RGP produce more deep layer neurons, it would produce less superficial layer neurons; and vice versa. This constraint is consistent with a defined or unitary neuronal output of individual RGPs, as previously shown [27]. While the average ratio of the deep versus superficial layer neurons across all clones is close to 1 (Fig. S3C), the preferred or predominant neuronal output ratio to either the deep or superficial layers at the clonal level is ∼2.2 (Fig. S3D). These results suggest that, while individual RGPs exhibit a preferred neuronal output to either deep or superficial layers, the total neuronal output of RGPs as a population is balanced to similarly support the formation of both deep and superficial layers.

Interestingly, we also observed a significant anti-correlation between the numbers of neurons in adjacent layers within individual clones labeled at E12, e.g. L6 versus L5, L5 versus L4, or L4 versus L2/3, but not between the numbers of neurons in non-adjacent layers, e.g. L6 versus L4 or L2/3, or L5 versus L2/3 (Fig. S3E–G). Moreover, this anti-correlation was specific for the experimental clone dataset, as no significant anti-correlation was found in the simulated dataset with the same average number of neurons per ‘clone’ and the same numbers of neurons in each layer as the experimental clonal dataset (Fig. S3H, I). Together, these results suggest that the neuronal outputs to different layers by individual RGPs are constrained and balanced at a fine level, which permits the variabilities, and at the same time, supports the development of all layers.

**Figure 2.**
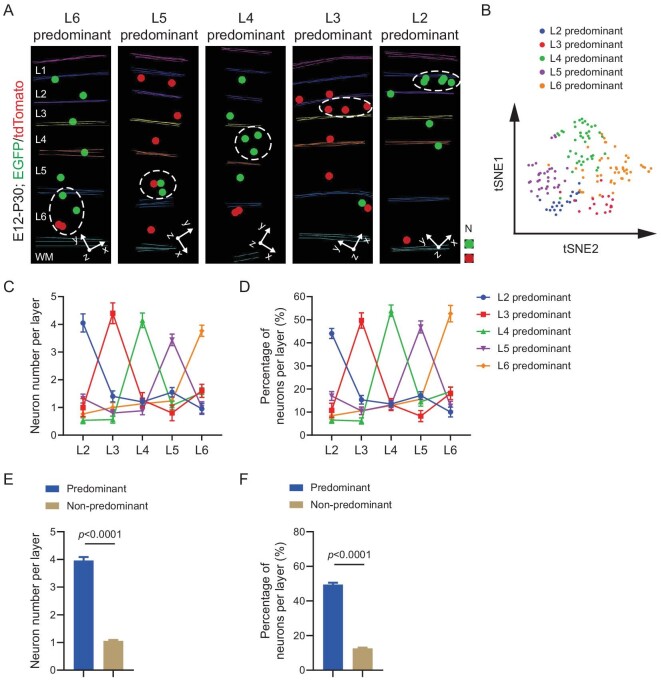
Consistent predominant neuronal layer outputs of individual RGPs to variable layers. (A) 3D reconstruction images of the representative layer-predominant clones labeled at E12. Colored lines indicate the layer boundary and colored dots represent the cell bodies of labeled neurons. L, layer; WM, white matter; N, neuron. (B) tSNE analysis of the clonal neuronal layer ratios. Neuron numbers in L2-6 were used as a 5-dimentional array for the analysis. A tSNE analysis was applied on the Principal Component Analysis (PCA) result of the clonal neuronal layer ratios (*n* = 167). Different colors indicate the clusters of clones exhibiting the predominance in corresponding layers. (C) Neuron numbers per layer for individual clones with different predominant layer neuronal outputs (L2, *n* = 20; L3, *n* = 15; L4, *n* = 41; L5, *n* = 25; L6, *n* = 30). The dot plots and error bars represent mean ± SEM. Clones exhibiting a predominance in L2–6 are presented in different colors. (D) Percentages of neurons per layer for individual clones with different predominant layer neuronal outputs (L2, *n* = 20; L3, *n* = 15; L4, *n* = 41; L5, *n* = 25; L6, *n* = 30). The dot plots and error bars represent mean ± SEM. Clones exhibiting a predominance in L2–6 are presented in different colors. (E) Neuronal numbers in the predominant and non-predominant layers (Predominant layers, *n* = 143; Non-predominant layers, *n* = 572). Bar plots and error bars represent mean ± SEM. (F) Percentages of neurons in the predominant and non-predominant layers (Predominant layers, *n* = 143; Non-predominant layers, *n* = 572). Bar plots and error bars represent mean ± SEM.

We further analyzed E12 labeled clones in the sensory cortices with all five layers of excitatory neurons (L2-6). Notably, we frequently observed clones with substantially more neurons in one particular layer than in other individual layers (Fig. 2A). Moreover, while the clones (i.e. the corresponding RGPs) were all labeled at E12, the predominant neuronal output layer varied, altering systematically from the deep to superficial layer. T-distributed Stochastic Neighborhood Embedding (t-SNE) analysis of the neuronal layer ratio for individual clones revealed five clusters with distinct predominant layer outputs (Fig. 2B). We quantified the number and percentage of neurons in individual layers for individual clones, and found that the clonal neuronal outputs to the predominant and non-predominant layers were highly consistent, even though the exact predominant layer identities for individual clones were different (Fig. 2C, D). The average number of neurons in the predominant layer regardless of the actual layer identity was ∼4, representing ∼50% of the total clonal neuronal output, whereas the average number of neurons in the non-predominant layer was ∼1, accounting for ∼10%–15% of the total clonal neuronal output (Fig. 2E, F). Together, these results demonstrate that individual RGPs display a highly consistent neuronal layer output pattern, with ∼50% to a particular layer as the predominant output and ∼10%–15% to other individual layers. At the same time, the predominant neuronal output layer for individual RGPs systematically varies to cover all five layers to ensure the effective assembly of the neocortex. Consistent with the sequential generation of deep to superficial layer neurons, the proportions of the deep layer predominant clones progressively decreased, whereas the proportions of the superficial layer predominant clones gradually increased, as the MADM labeling time shifted from the early to late developmental stage (Fig. S4A).

### IP generation contributes to predominant layer neuronal output

RGPs divide to produce neurons either directly or indirectly via IPs (i.e. direct or indirect neurogenesis) (Fig. S4B). Compared with direction neurogenesis, indirect neurogenesis not only increases the neuronal output, but also affects the layer positioning of neurons that is coupled to their birthdate and the subtype specification and functional organization [45,46]. Additional round(s) of cell division via IPs would postpone the actual birthdate of neurons upon RGP division and consequently influence their layer occupation [40,45,47]. In other words, indirect neurogenesis via an IP gives rise to neurons with a similar birthdate to neurons generated by the original RGP at the subsequent division, resulting in more neurons to occupy a similar layer. We thus tested whether IP generation is linked to the predominant neuronal layer output by RGPs. To assess IP generation, we utilized the unique resolution of MADM labeling that allows distinguishing the two daughter cells of the first division of the labeled RGP, as well as their progenies explicitly. Should the number of the minority color labeled neurons in a clone be one, it indicates a direct neurogenic division to produce a neuron (Fig. S4C, left). On the other hand, should the number of the minority color labeled neurons in a clone be two or more, it indicates an indirect neurogenic division to produce an IP that divides further to produce two or more neurons (Fig. S4C, right). By systematically analyzing the numbers of the minority color labeled neurons in clones labeled at different developmental stages, we assessed the fractions of direct versus indirect neurogenesis across the neurogenic phase (Fig. S5A), which appeared to be consistent with the mitotic capacity of TBR2^+^ IPs in the SVZ (Fig. S5B–E). Moreover, most of the minority color labeled two neurons in individual clones were located in the same layer (Fig. S4D).

**Figure 3.**
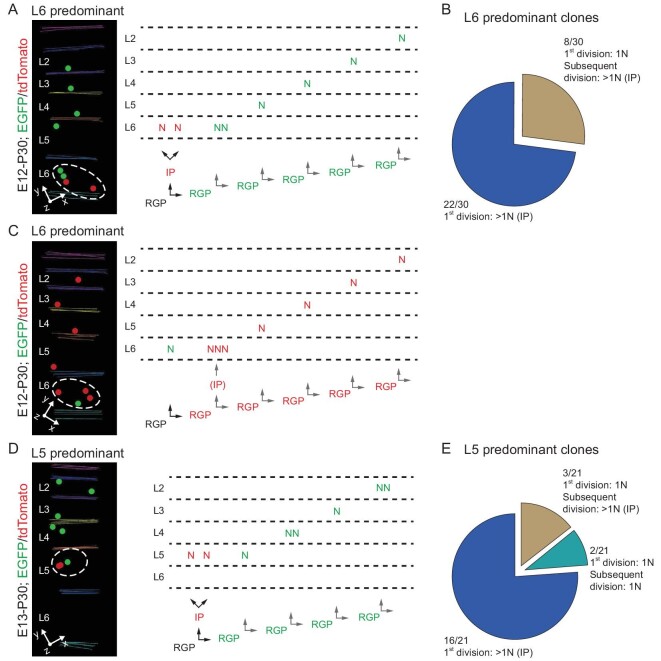
Association between IP generation and predominant layer neuronal output. (A) 3D reconstruction image of a representative L6 predominant clone labeled at E12. Inferred lineage tree of the clone is shown to the right. In the first division, the labeled RGP (black) generates an IP (red) and an RGP (green) through an asymmetric division, and subsequently the IP (red) generates two red neurons located in L6; the renewed RGP (green) continues to undergo asymmetric divisions to generate six neurons (green) distributed in L6-2. Colored lines indicate the layer boundary and colored dots represent the cell bodies of labeled neurons. The neurons in the predominant layer (L6) are circled by the white broken line. (B) The ratio of direct and indirect neurogenesis in L6 predominant RGP clones (*n* = 30). Note that clones underwent direct neurogenesis at the 1st division produce more than 1 neuron in L6, indicating IP generation and indirect neurogenesis. (C) 3D reconstruction image of a representative L6 predominant clone labeled at E12. Inferred lineage tree of the clone is shown to the right. In the 1st division, the labeled RGP (black) generates a neuron (green) and an RGP (green) through an asymmetric division; then the renewed RGP (green) continues to undergo asymmetric divisions to generate seven neurons (red) with three distributed in L6-2. Neurons in the predominant layer (L6) are circled by the white broken line. (D) 3D reconstruction image of a representative L5 predominant clone labeled at E13. Inferred lineage tree of the clone is shown to the right. In the 1st division, the labeled RGP (black) generates an IP (red) and an RGP (green) through an asymmetric division, and the IP (red) subsequently divide to generate two neurons (red) distributed in L5; Moreover, the renewed RGP (green) continues to undergo asymmetric divisions to generate six neurons (green) distributed in L5-2. The neurons in the predominant layer (L5) are circled by the white broken line. (E) The ratio of direct neurogenesis versus indirect neurogenesis in L5 predominant RGP clones (*n* = 21).

We focused on E12 labeled clones and assessed the contribution of IP generation or indirect neurogenesis to the predominant layer neuronal output. In this example a clone with a predominant neuronal output to layer 6 (Fig. 3A), the first division produced two minority color (i.e. red) labeled neurons located in layer 6, indicating that IP generation and the subsequent indirect neurogenesis contribute to the predominant layer output in layer 6. Strikingly, amongst 30 clones with a predominant layer 6 neuronal output, 22 clones exhibited IP generation and indirect neurogenesis to generate layer 6 neurons in the first division (Fig. 3B). In the remaining 8 clones, while the first division was a direct neurogenesis with a single minority color labeled neuronal output in layer 6, more than one neuron in the majority color were found in layer 6 (Fig. 3C), indicating that the subsequent division of the labeled RGP produces layer 6 neurons via IP generation (Fig. 3B). These results suggest that IP generation and indirect neurogenesis contribute to the predominant layer 6 neuronal output in individual clones.

Similarly, in E13 labeled clones with a predominant neuronal output to layer 5, more than 75% of clones (16 out of 21) displayed IP generation and indirect neurogenesis for producing neurons in layer 5 (Fig. 3D, E), indicative of a link between IP generation and predominant layer 5 neuronal output. Taken together, these results suggest that IP generation and indirect neurogenesis are associated with the predominant layer neuronal output by individual RGPs.

### Disruption of IP generation impairs predominant layer neuronal output

**Figure 4.**
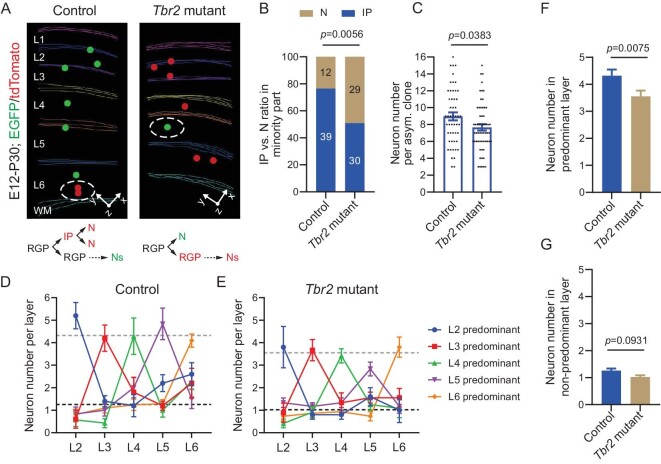
*Tbr2* deletion impairs the predominant layer neuronal output by RGPs. (A) 3D reconstruction images of the representative P30 control and *Tbr2* mutant clones labeled at E12. Colored lines indicate the layer boundary and colored dots represent the cell bodies of labeled neurons. The minority color labeled neurons reflecting neuron (direct neurogenesis) versus IP (indirect neurogenesis) production are circled by white broken lines. (B) Direct (N) and indirect neurogenesis (IP) ratios of the control (*n* = 51) and *Tbr2* mutant clones (*n* = 59). The numbers of clones are shown in the plot. Statistical analysis was performed using Chi-square test. (C) Quantification of the number of neurons in the control (*n* = 51) and *Tbr2* mutant clones (*n* = 59). Bar plots and lines represent mean ± SEM and dots represent individual clones. Statistical analysis was performed using two-sided Mann–Whitney–Wilcoxon test. (D) Neuron numbers per layer in the control clones (L2, *n* = 8; L3, *n* = 6; L4, *n* = 8; L5, *n* = 10; L6, *n* = 29). The dot plots and error bars represent mean ± SEM. Colors indicate clones with different predominant layer outputs in L2-6. (E) Neuron numbers per layer in the *Tbr2* mutant clones (L2, *n* = 12; L3, *n* = 10; L4, *n* = 12; L5, *n* = 9; L6, *n* = 19). The dot plots and error bars represent mean ± SEM. Colors indicate clones with different predominant layer outputs in L2-6. (F) Neuron numbers in the predominant layer per clone in the control (*n* = 44) and *Tbr2* mutant clones (*n* = 47). Statistical analysis was performed using two-sided Mann–Whitney–Wilcoxon test. (G) Neuron numbers in the non-predominant layer per clone in the control (*n* = 176) and *Tbr2* mutant clones (*n* = 188). Statistical analysis was performed using two-sided Mann–Whitney–Wilcoxon test.

To further assess the link between IP generation and the predominant layer neuronal output, we took advantage of the mutant allele of *Tbr2* [48], which encodes a transcription factor preferentially expressed in IPs, and combined it with *Emx1-CreER^T2^; MADM-11* (Fig. S6A). Individual neocortical excitatory neuron clones were labeled at E12 and examined at P30 as described above (Figs S6B and 4A). As expected, compared with the control clone, the *Tbr2* mutant clone showed a significant decrease in IP generation in the first division (Fig. 4A, B). Notably, IP generation and indirect neurogenesis were not completely eliminated upon *Tbr2* deletion, consistent with previous studies [49,50]. The average number of neurons in the *Tbr2* mutant clone was significantly smaller than that of the control clone (Fig. 4C), consistent with a loss of IP. Interestingly, we observed a systematic reduction in the predominant layer neuronal output in the *Tbr2* mutant clone compared with the control clone (Fig. 4D, E). As a result, the average number of neurons in the predominant layers of the *Tbr2* mutant clone was significantly smaller than that of the control clone (Fig. 4F), whereas the average number of neurons in the non-predominant layers remained largely comparable between the control and *Tbr2* mutant clones (Fig. 4G). These results suggest that IP generation and indirect neurogenesis preferentially contribute to the predominant layer neuronal output.

### Variable but patterned neuronal layer output of individual RGPs

**Figure 5.**
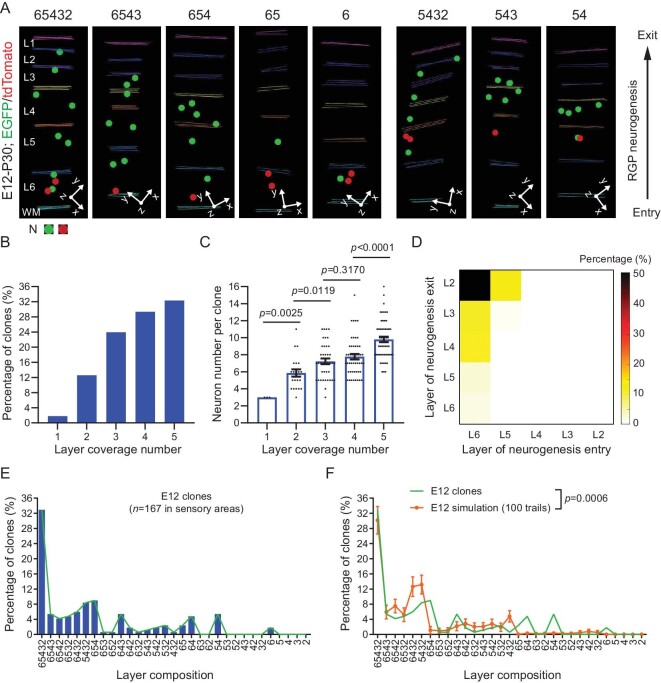
Clonal variability in neuronal layer composition reflecting neurogenic entry and exit timing differences of RGPs. (A) 3D reconstruction images of the representative P30 clones in the sensory areas labeled at E12 with different layer compositions. L, layer; WM, white matter; N, neuron; The numbers at the top indicate layer compositions of individual clones. (B) Percentage of clones (*n* = 167) with different numbers of layer coverage. (C) Neuron numbers of the clones with different layer coverage numbers (1-layer, *n* = 3; 2-layer, *n* = 27; 3-layer, *n* = 49; 4-layer, *n* = 54; 5-layer, *n* = 57). Note that the more layer coverage number, the larger the neuron number per clone. Bar plots and error bars represent mean ± SEM and dots represent individual clones. Statistical analysis was performed using two-sided Mann–Whitney–Wilcoxon test. (D) Heatmap of the percentage of clones (*n* = 167) with different neurogenic entry and exit timings. Colors indicate the percentage of clones. Note that the majority of E12 labeled clones enter neurogenesis to produce neurons in L6 and exit neurogenesis to produce neurons in L2. A fraction of clones exit neurogenesis earlier to produce the last neuronal productions in L3 and L4. In addition, a fraction of clones enter neurogenesis earlier to produce neurons in L5 at E12. (E) Layer composition distributions of E12 labeled clones (*n* = 167) in the sensory cortices. Bars reflect the percentage of the specific clones with corresponding layer compositions. The green line indicates the trend of the distribution. (F) Distinct layer composition distributions of E12 labeled clone dataset (*n* = 167, green line) and the simulated dataset (*n* = 167 for 100 trails, orange line with error bars). Lines and error bars represent mean ± SEM. Statistical analysis was performed using Chi-square test.

To further dissect RGP neuronal output and neocortex assembly, we next examined the precise neuronal layer output of individual RGPs. We focused on E12 labeled clones in the sensory cortices with all five excitatory neuronal layers (L2-6) and quantitatively assessed the layer composition of individual clones at P30 (Fig. 5). We observed many clones containing neurons in all five layers (Fig. 5A, left). On the other hand, we also observed clones lacking neurons in one or more layers (Fig. 5A, right). Notably, the more layers a clone covered, the more neurons it contained (Fig. 5B, C), indicating that the layer coverage is generally linked to the neuronal generation capacity of RGPs.

RGPs produce neurons via asymmetric divisions, during which RGPs are renewed for the next round of division and, at the same time, neurogenesis occurs. Should an RGP fail to renew itself (e.g. exit the cell cycle by committing a terminal division), neurogenesis is terminated, resulting in a lack of subsequent neuronal generation. Based on the birthdate-dependent radial migration and layer occupation, the timings of a particular RGP as it enters and exits the neurogenic phase would affect its neuronal output and layer occupation. For example, if an RGP enters the neurogenic phase earlier than E12, MADM labeling at E12 would miss the labeling of the earliest born neuron(s) locating in the deep layer 6. On the other hand, if an RGP exits the neurogenic phase earlier, there would be fewer late born neurons occupying the superficial layer(s). Consistent with this, in addition to the local adjacent layer anti-correlations linked to IP generation, the clonal variabilities in neuronal layer occupation largely reflected the variations in the timings of RGPs entering and exiting the neurogenic phase (Fig. 5A, D, E). The majority of RGPs entered the neurogenic phase to produce layer 6 neurons at E12 and exited the neurogenic phase after generating layer 2 neurons (Fig. 5A, D). Yet, in alignment with an earlier cell cycle or neurogenic exit, some clones generated layer 3, 4, 5, or 6 neurons as the last neuronal output (Fig. 5A, D). In addition, some clones generated layer 5 neurons at E12, largely indicating an earlier neurogenic entry (Fig. 5A, D). Together, these results suggest that individual RGPs undergo consecutive asymmetric cell divisions to generate neurons progressively to occupy the deep to superficial layers with some variabilities in the timings of entering and exiting the neurogenic phase, leading to the corresponding variations in neuronal layer output.

We systematically analyzed the neuronal layer composition of all E12 labeled clones in the sensory cortices (Fig. 5E). We found that more than 30% of clones contained neurons in all five layers. The remaining clones exhibited the layer compositions largely reflecting the earlier exiting or entering of the neurogenic phase. In addition, the balanced and constrained neurogenesis of adjacent layers with significant anti-correlations may also lead to skipping or missing neurons in a particular layer (e.g. L6542, L6432). Notably, we rarely observed any clones skipping or missing neurons in two adjacent layers in between (e.g. L632, L52). Importantly, the layer composition pattern of the experimental clonal dataset was significantly different from that of a simulated dataset with the same average neuron number per ‘clone’ and the same numbers of neurons in individual layers as the experimental clonal dataset (Fig. 5F). Together, these results suggest that the neuronal layer output of individual RGPs is not random, but constrained and patterned with defined variabilities.

### Predictable neuronal layer output of RGPs

**Figure 6.**
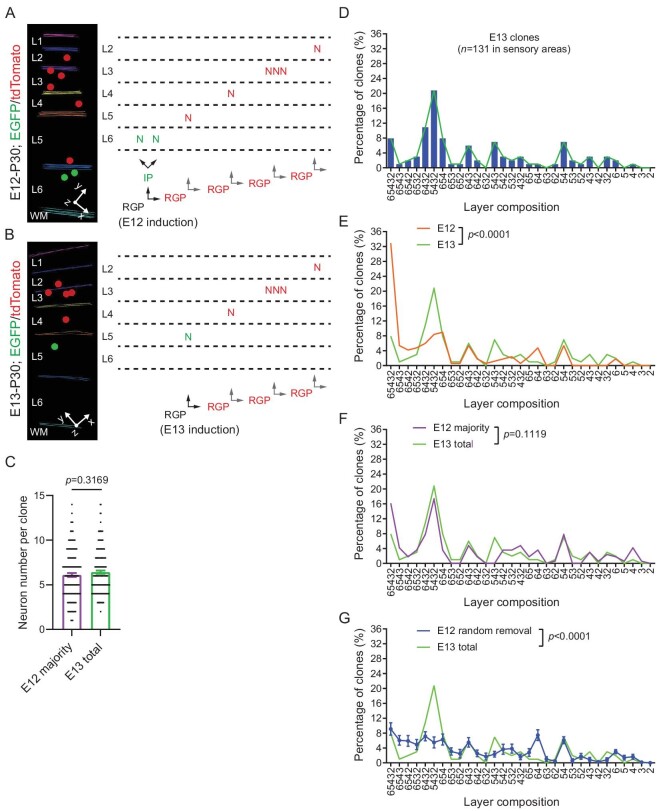
Predictable neuronal layer composition patterns of individual RGPs. (A) 3D reconstruction image of a representative clone labeled at E12. Inferred lineage tree of the clone is shown to the right. The originally labeled RGP (black) divides and generates an IP (green) and an RGP (red) at the 1st division; the IP (green) continues to divide and generates two neurons (green) located in L6; the renewed RGP (red) continues to undergo asymmetric divisions and generates 6 neurons (red) distributed in L5-2. Colored lines indicate the layer boundary and colored dots represent the cell bodies of labeled neurons. (B) 3D reconstruction image of a representative clone labeled at E13. Inferred lineage tree of the clone is shown to the right. The originally labeled RGP (black) divides to generate a neuron (green) and an RGP (red); the renewed RGP (red) continues to undergo asymmetric divisions to generate 5 neurons distributed in L4-2. Colored lines indicate the layer boundary and colored dots represent the cell bodies of labeled neurons. (C) Neuron numbers of the majority color part of E12 clones (E12 majority, *n* = 167) and the total E13 clones (E13 total, *n* = 184). Statistical analysis was performed using two-sided Mann–Whitney–Wilcoxon test. (D) Layer composition distribution of E13 labeled clones (*n* = 131) with corresponding layer compositions. Bars reflect the percentage of clones with different layer compositions. The green line indicates the trend of the distribution. (E) Distinct layer composition distributions of E13 (*n* = 131) and E12 (*n* = 167) labeled clones. Statistical analysis was performed using Chi-square test. (F) Similar layer composition distributions of E13 labeled clones (*n* = 131) and the majority part of E12 labeled clones (*n* = 167). Statistical analysis was performed using Chi-square test. (G) Distinct layer composition distributions of E13 labeled clones (*n* = 131) and E12 labeled clones with a random removal of the same number of neurons as the minority part (*n* = 167 for 100 repeats). Statistical analysis was performed using Chi-square test.

To further test that the neuronal layer output of individual RGPs is constrained and patterned, we next asked whether the neuronal layer output of RGPs

is predictable. We took advantage of the resolution of MADM labeling in distinguishing daughter cell lineages and quantitatively compared the neuronal layer output of individual RGPs labeled at E12 and E13 (Fig. 6). In MADM-labeled asymmetric neurogenic clones, the majority color labeled neurons reflect the neuronal output of the renewed RGP of the first division, whereas the minority color labeled neurons represent the neuronal output of the first neurogenic division by the originally labeled RGPs. Indeed, we found that the number of the majority color labeled neurons in E12 labeled clones was the same as the total number of neurons in E13 labeled clones (Fig. 6A–C).

Next, we quantitatively examined the neuronal layer composition of individual E13 labeled clones, which exhibited clear variabilities (Fig. 6D). Notably, distinct from E12 labeled clones with the predominant layer composition including all layers (i.e. L65432), the most abundant layer composition of E13 labeled clones included layers 5, 4, 3, and 2, but lacked layer 6 (i.e. L5432), consistent with a relatively late MADM labeling time from E12 to E13.

E13 labeled clones also exhibited clear variabilities in the layer composition reflecting the timing variations of individual RGPs entering and exiting the neurogenic phase. As expected, the layer composition pattern of E13 labeled clones was significantly different from that of E12 labeled clones (Fig. 6E). However, if we selectively focused on the majority color labeled neurons in E12 labeled clones, their layer distribution pattern was highly similar to that of E13 labeled clones (Fig. 6F). These results suggest that the layer composition of E13 labeled clones can be inferred or predicted from E12 labeled clones, arguing against a random neuronal layer output of RGPs. Moreover, we found that a random removal of the same number of neurons as the minority color labeled neurons in E12 labeled clones did not lead to a similar neuronal layer composition pattern to that of E13 labeled clones (Fig. 6G). Together, these results suggest that the neuronal output layer composition of RGPs is predictable and coupled to the orderly RGP division.

## DISCUSSION

The assembly of the neocortex represents one of the most intricate processes in developmental biology. From the view of the formation of a complex system, reliability and robustness are two essential aspects to implement and balance. Reliability reflects the ability of the process to properly produce the complex system, whereas robustness indicates the degree of the process that tolerates variations. High reliability often entails a rigorous control with a low tolerance to variations or less robustness. On the other hand, high robustness is typically associated with large variabilities and high reliability uncertainty. Therefore, balanced reliability and robustness is crucial for the effective assembly of a complex system. Yet, how reliability and robustness is achieved and balanced in the assembly of the complex neocortex remains largely unclear. In this study, by systematically and quantitatively analyzing the neuronal output of individual RGPs, the predominant building blocks of the neocortex, we found that RGPs undergo stable asymmetric divisions to produce neurons at a largely steady rate to ensure a reliable neuronal production for neocortical assembly. At the same time, individual RGPs display systematic and constrained variabilities in neuronal layer output linked to IP generation to support a robust formation of all layers. Therefore, reliability and robustness in the assembly of the complex neocortex is implemented and balanced in the fundamental processes of neurogenesis (Fig. S7).

As the predominant neural progenitors, RGPs undergo consecutive asymmetric neurogenic divisions to produce diverse neurons in a progressive manner. We performed MADM labeling of individual RGPs systematically every 24 hours across the entire neurogenesis period (i.e. E12–E16) in the developing mouse neocortex and quantitatively analyzed their neuronal outputs, with regard to neuronal number and layer occupation, the two key aspects of neocortical assembly. By analyzing the clonal neuronal number histogram at each neurogenic stage, we found that the clonal neuronal number variabilities can be well-approximated by a common Poisson distribution. More strikingly, the means of the Poisson distributions at different neurogenic stages exhibit a linear relationship, in which the y-axis intercept reflecting the mean total number of neuronal outputs by an RGP across the entire neurogenesis period is ∼9, and the slope or the mean rate of neurogenesis is ∼1.6 neurons per day. Notably, the fraction of excitatory neuron cell death in the developing neocortex is relatively small and is unlikely to contribute to any major underestimation of RGP neuronal output [51,52]. These results suggest that the neurogenesis process conducted by RGPs is rather steady and reliable despite some variabilities. This stable and reliable process of neurogenesis by RGPs serves as the primary backbone of neocortical assembly. The symmetric proliferative phase prior to the neurogenic phase leads to a summation of individual neurogenic units in size and layer output.

The mean RGP neuronal output number of ∼9 is consistent with previous studies [27,33]. It further supports that the neurogenic potential of individual RGPs is constrained at a defined number. As a reflection of this constraint, we observed interesting neuronal layer output patterns in individual clones. In particular, the clonal neuronal outputs to the deep versus superficial layers exhibit a clear anti-correlation, which points to two important features of RGP neurogenesis. On one hand, the total neuronal output by individual RGPs is relatively defined; on the other hand, the exact neuronal layer output can be variable. This balanced reliability and variability allows individual RGPs to have variable neuronal outputs and, at the same time, this variability is constrained to support both the superficial and deep layer formation. Importantly, similar anti-correlated and balanced neuronal output patterns were also observed for adjacent layers, but not for non-adjacent layers. These results further suggest that the neurogenesis process by RGPs is constrained and balanced to allow reliability and variability, even at the fine levels of individual layers.

Notably, this balanced reliability and variability in RGP neurogenesis can also be observed at another level. We found that individual RGP clones often exhibit a preferred or predominant neuronal layer output with ∼50% of neurons in one particular layer. This predominant neuronal layer output is remarkably consistent and reliable for RGPs; yet, the exact layer of the predominant neuronal output varies systematically to cover both deep and superficial layers, thereby ensuring an effective assembly of all layers. Interestingly, this consistent and reliable generation of a predominant neuronal output to variable layers by RGPs depends on IP production and indirect neurogenesis, which represent a crucial mechanism for the increase of neurogenesis capacity and neocortical expansion. However, the mechanism by which IP-mediated indirect neurogenesis contributes to different predominant layers for RGPs at the same developmental stages (e.g. labeled at E12) remains to be explored.

Our data suggest that at the individual RGP level, the IP generation does not occur at every round of RGP asymmetric division; instead, it occurs only at some rounds of RGP asymmetric division, leading to the output of the preferred or predominant neuronal layer. Interestingly, the exact round(s) of RGP asymmetric division with IP generation for individual RGPs appear variable, resulting in different predominant neuronal layer outputs. These results suggest that IP generation by individual RGPs exhibits a constrained degree of variability. While some RGPs produce IPs and consequently more neurons in the deep layers, others produce IPs and more neurons in the superficial layers. Notably, during RGP neurogenesis, how the direct versus indirect neurogenesis is determined at each round of asymmetric division remains largely unclear. It is possible that IP generation depends on critical fate-determining factors with oscillatory and accumulative expression patterns, similar to RGP or neuronal fate determination processes [26,53]. When their expression reaches a threshold, an IP is generated; otherwise, a neuron is generated. Moreover, upon an IP generation, the expression of the IP fate-determining factors is reset for the next round of oscillation and accumulation. Under these conditions, different RGPs reach the IP fate specification threshold at different developmental stages, leading to variable predominant neuronal layer outputs.

Besides the predominant layer, individual RGPs also generate neurons located in other layers. In fact, the exact layer composition of individual clones exhibits clear variabilities. A previous study suggested that the neuronal output layer composition of individual RGPs is stochastic [33]. Notably, our data indicated that the neuronal layer composition of individual RGP clones is patterned and predictable, which accounts for the majority of clonal layer composition variabilities observed experimentally. In particular, more than 30% of RGP clones labeled at E12 contained neurons in all five layers. The remaining clones display a variable combination of neurons in different layers. However, the variabilities largely reflect IP generation, as well as the timing differences of RGPs entering and exiting the neurogenic phase. The pseudostratified organization of RGPs dictates that their cell cycle and lineage progression are not all synchronous [54]. While the neurogenic phase of RGPs in the developing mouse neocortex occurs largely between E12 and E16, not all RGPs enter the neurogenic phase precisely at E12 or exit at E16. For RGPs that enter the neurogenic phase earlier than E12, MADM labeling at E12 would miss the neurons generated prior to E12 in the deep layers. Similarly, for RGPs that exit the neurogenic phase earlier than E16, their neuronal output would lack neurons generated at the later stage. The other important source of neuronal layer distribution variabilities is the generation of the anti-correlated adjacent neuronal layer, which may lead to a lack or skip of neurons in one layer. We rarely observed RGP clones skipping neurons in two adjacent layers in between, in line with the observation of no anti-correlation between the numbers of neurons in non-adjacent layers. These findings suggest that the neuronal layer output by individual RGPs is built on the stable backbone of RGP asymmetric division as well as IP generation. Indeed, we found that the neuronal layer output by individual RGPs at the late developmental stage (e.g. E13) can be predicted at the early developmental stage (e.g. E12), further indicating that the neuronal layer output by RGPs is regulated with constrained patterns and variabilities. While one cannot completely rule out any contribution of stochastic processes in neocortical neurogenesis, recent studies suggest that developmental origin and lineage history influence the fine neuronal subtype specification and functional organization [46,55,56], further implying that the generation of diverse neurons with distinct features is highly regulated.

In summary, our study demonstrates that the complex yet organized neocortex is assembled on the basis of a stable framework of RGP asymmetric division, which ensures a steady production of neurons, and a variable but constrained IP generation, which allows systematic variabilities in neuronal number and layer occupation, in conjunction with the birthdate-dependent inside-out neuronal migration and positioning. This combination of a stable RGP asymmetric division and a variable but constrained IP generation allows a balance of reliability and robustness in neuronal production for the effective construction of the complex neocortex.

## MATERIALS AND METHODS

### Mouse lines

*MADM-11GT* (stock# 013749) and *MADM-11TG* (stock# 013751) mouse lines [38] were obtained from Jackson Laboratory (Bar Harbor, ME); *Emx1-CreER^T2^* [37] and *Tbr2^fl/^^fl^* [57] mouse lines were kindly provided by Dr. Nicoletta Kessaris and Dr. Anna-Katerina Hadjantonakis, respectively. CD-1 mice were obtained from Beijing Vital River Laboratory Animal Technology Co., Ltd (Beijing, China). Genotyping was carried out using standard PCR protocols. Both male and female mice were used in the study. For clone induction at the embryonic stage, pregnant females were either injected intraperitoneally (E12, E13 and E14: 10–20 μg/g of body weight) or orally gavaged (E15, E16: 200–400 μg/g of body weight) with Tamoxifen (T5648, Sigma) dissolved in corn oil (C8267, Sigma). Live embryos were recovered at E18–E19 through cesarean section, fostered, and raised for further analysis. The mice were maintained at the animal facility of Tsinghua University, and all animal procedures were approved by the Institutional Animal Care and Use Committee (IACUC). For timed pregnancies, the plug date was designated as E0 and the date of birth was defined as P0. No wild animal or field-collected sample was used in the study.

### Brain sectioning, immunohistochemistry, imaging, and 3D reconstruction

Mice were perfused intracardially with ice-cold phosphate-buffered saline (PBS, pH 7.4), followed by 4% paraformaldehyde (PFA) in PBS (pH 7.4). Brains were post-fixed with 4% PFA at 4°C for ∼6 hours, cryo-protected, and sectioned at 20, 60 or 100 μm using cryostat (for embryonic brains) or microtome (for adult brains) (Leica Biosystems) for immunohistochemistry, as previously described [27]. The following primary antibodies were used: chicken anti-GFP (GFP-1020; 1:1000; Aves), rabbit anti-RFP (600-401-379; 1:500; Rockland), rat anti-TBR2 (14-4875-82; 1:100, ThermoFisher), rabbit anti-P-HH3 (ab47297; 1:1000, Abcam), mouse anti-Ki67 (556 003; 1:200; BD Pharmingen). Alexa flour 488-Donkey anti-chicken (703-546-155; 1:1000; Jackson ImmunoResearch), Alexa flour 555-Donkey anti-rabbit (A31572; 1:1000; ThermoFisher), and Alexa flour 488-Donkey anti-rat (A-21208; 1:1000; ThermoFisher) secondary antibodies were used.

Brain sections were mounted on glass slides, imaged using confocal microscopy (FV3000, Olympus) or slide scanner (Axio Z1, Zeiss), and reconstructed using Neurolucida (MBF Bioscience). For 3D reconstruction, each section was analyzed sequentially in the rostral to caudal order. The boundaries of the entire brain and lateral ventricles were traced and aligned. Individual labeled neurons, astrocytes, and oligodendrocytes were represented as colored symbols (three to four times the size of the cell body). Layer boundaries based on nuclear staining were also documented. Cortical areas were identified based on the Allen Brain Atlas (http://mouse.brain-map.org/static/atlas). Images were analyzed by ZEN (Zeiss), FV31S-DT (Olympus), IMARIS (Bitplane), or Photoshop (Adobe).

### Quantification and statistical analysis

Mice subjected to the analyses were littermates, age-matched, and include both sexes. Sample size was determined to be adequate based on the magnitude and consistency of measurable differences between groups. Statistical significance was determined using Chi-square or two-sided non-parametric Mann–Whitney–Wilcoxon test or Kolmogorov–Smirnow test, and the test results were given as exact values in the figures. Statistical significance was defined as *p* < 0.05. Statistical tests were performed with Prism (version 7, GraphPad). Effect sizes were calculated using Pearson's r (Chi-square) or U/(n1*n2) (Mann–Whitney–Wilcoxon test). Values in bar graphs indicate mean ± SEM.

## Supplementary Material

nwad247_Supplemental_File
