# Supplementary material for: How variable progenitor clones construct a largely invariant neocortex
Source: Natl Sci Rev. 2023 Sep 15;11(1):nwad247. doi: 10.1093/nsr/nwad247 (PMC10810685; doi:10.1093/nsr/nwad247)
Supplement: nwad247_Supplemental_File [file nwad247_supplemental_file.pdf]

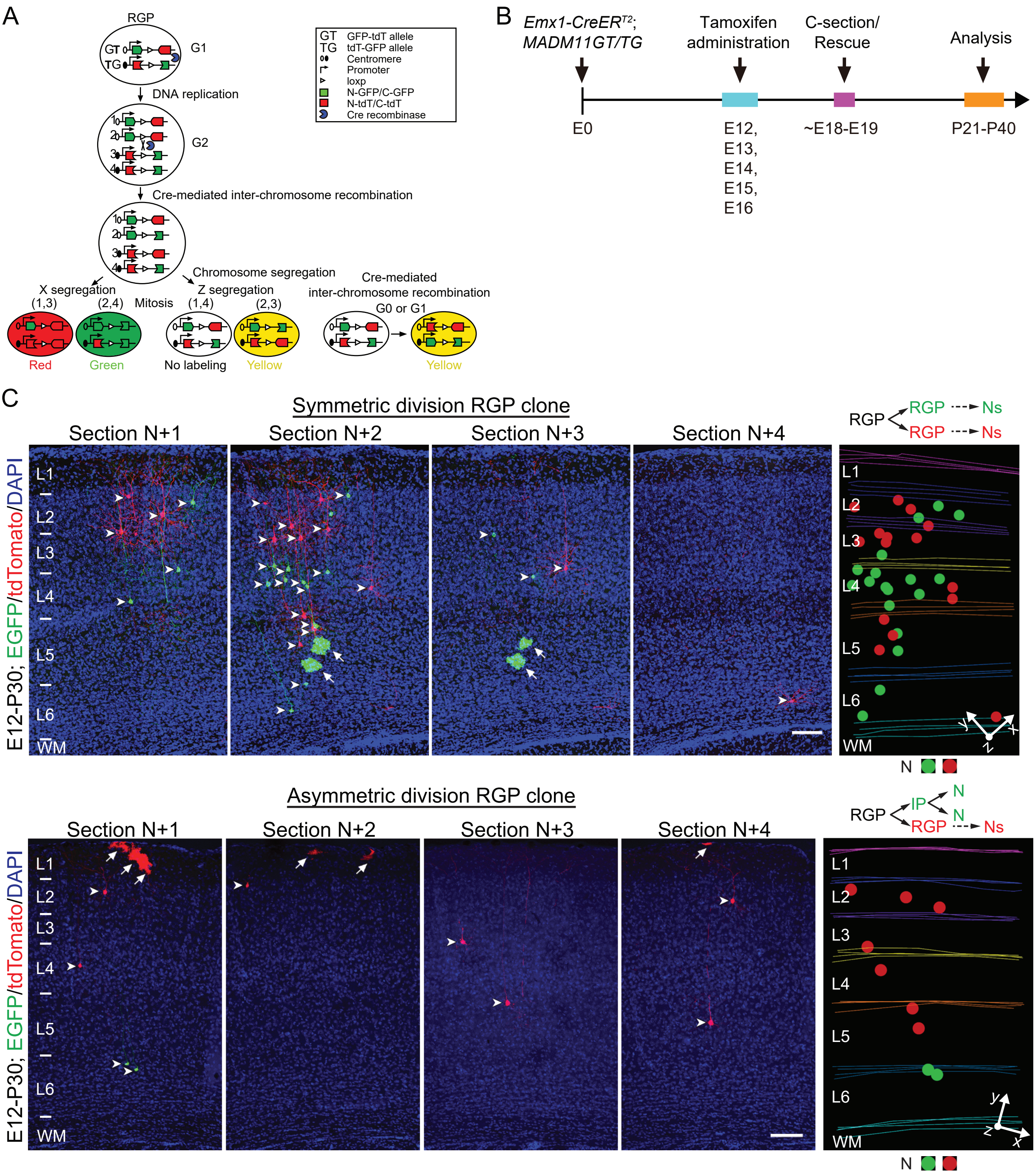

Shen et al., Figure S1

SUPPLEMENTAL FIGURE LEGENDS

Figure S1. MADM labeling of individual dividing RGPs in the developing mouse neocortex.

(A) Schematic diagram of MADM labeling strategy.

(B) Experimental diagram of MADM-based clonal analysis of neocortical neurogenesis across the entire neurogenic phase.

(C) Representative confocal images and 3D reconstruction images of RGP clones undergo symmetric (top) or asymmetric (bottom) division. Neurons (arrowheads) and glia (arrows) in individual clones are labeled by EGFP (green) and tdTomato (red). Different layers are annotated based on distinct histological features indicated by DAPI staining (blue). For 3D reconstruction images, colored lines indicate the layer boundary and colored dots represent the cell bodies of labeled neurons. The x/y/z axes indicate the spatial orientation of the clone with the y axis parallel to the midline and pointing dorsally. Scale bar: 200 μm.

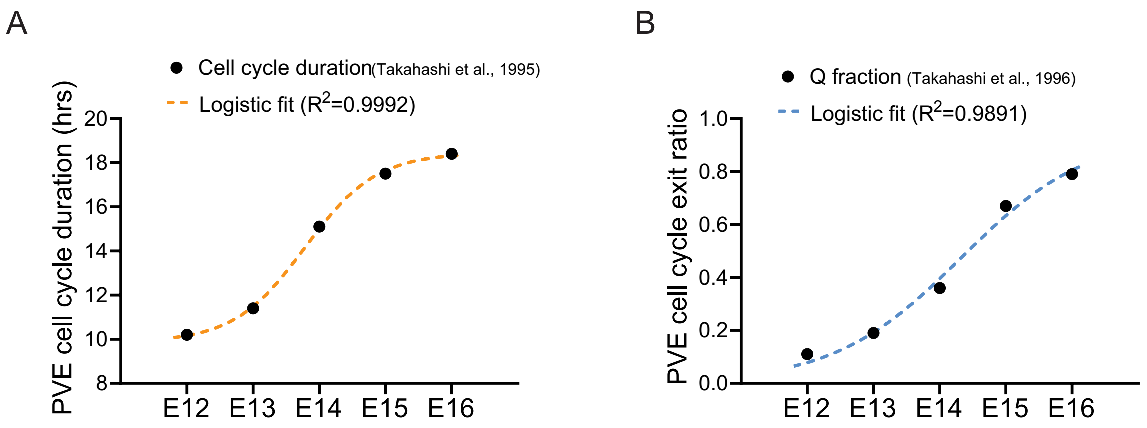

C

	E12	E13	E14	E15	E16
**Mean clone size (Exp.)**	7.73	5.81	3.95	2.68	1.68
Minority size (Exp.)	1.70	1.49	1.39	1.35	1.19
PVE cycle exit ratio	0.06	0.23	0.39	0.61	0.79
PVE cycle duration (hrs)	10.2	11.4	15.1	17.5	18.4
Daily division rounds	2.22	1.81	1.47	1.34	1.30
Daily output	3.55	2.62	2.02	1.70	1.56
Predicted clone size (w/o Q correction)	11.44	7.89	5.28	3.26	1.56
**Predicted clone size (w/ Q correction)**	8.05	5.50	3.64	2.54	1.56

$$\text{Ideal clone size } (day = X) = \sum_{day=X}^{E16.5} (\text{Minority size}_{day} * \text{Division rounds}_{day}), \quad X = E12.5, E13.5, \ldots$$

$$\text{Corrected clone size } (day = X) = \frac{1}{1 - Q_x} * \sum_{day=X}^{E16.5} (1 - Q_X) * (\text{Minority size}_{day} * \text{Division rounds}_{day}), \quad X = E12.5, E13.5, \ldots$$

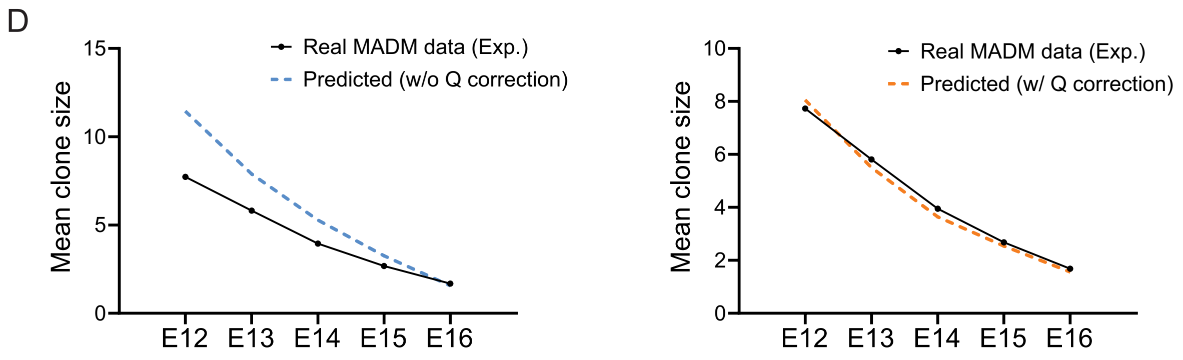

Shen et al., Figure S2

Figure S2. Estimation of clonal neuronal output of RGPs based on MADM clonal dataset and cell cycle dynamics.

(A) Cell cycle duration of the pseudostratified ventricular epithelial (PVE) cells (largely RGPs) at E12-E16 based on Takahashi et al., J Neurosci 1995[43]. The orange broken line represents a logistic fitting of the cell cycle duration change across different developmental stages.

(B) Cell cycle exit ratio (Q fraction) of PVE cells at E12-E16 based on Takahashi et al., J Neurosci 1996[44]. The blue broken line represents a logistic fitting of the Q fraction change across different developmental stages.

(C) Predictions of neuronal outputs of individual RGPs based on MADM clonal dataset and PVE cell cycle duration without or with correction of Q fraction. The estimation formula of the predicted clone size without or with Q correction are shown at the bottom.

(D) Comparison of MADM experimental clone sizes (Exp.) with predicted clone sizes without (left) or with (right) Q correction. Note that Q fraction correction faithfully predicts the size (i.e., neuron number) of MADM experimental clones.

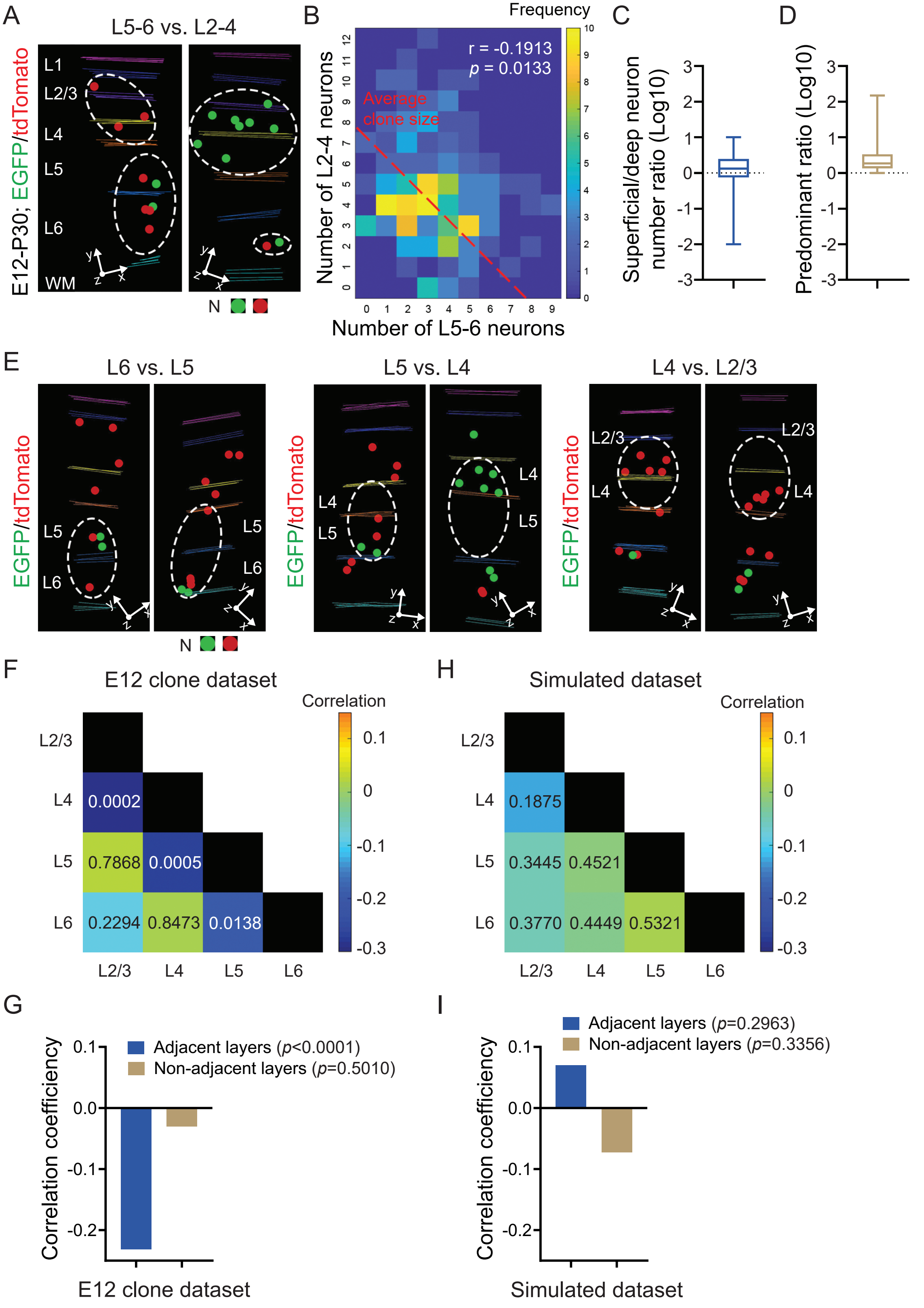

Shen et al., Figure S3

Figure S3. Constrained and balanced neuronal outputs to different layers by individual RGPs.

(A) 3D reconstruction images of the representative P30 clones with more neurons in the deep (L5-6, left) or superficial (L2-4, right) layers labeled at E12. Colored lines indicate the layer boundary and colored dots represent the cell bodies of labeled neurons. Neurons in the superficial and deep layers are circled with white broken lines, respectively.

(B) Heatmap of the correlation between the neuron numbers in the superficial (L2-4) and deep (L5-6) layers (n=167). The colors of heatmap represent the frequency of corresponding clones. The red broken line indicates the average clone size. Pearson correlation was applied to test the significance of the anti-correlation. Correlation coefficiency (r) and *p* value are shown in the inset.

(C) Ratio of neuron numbers in individual clones (n=167) in the superficial and deep layers.

(D) Ratio of neuron numbers in the predominant versus non-predominant group of the superficial and deep layers (n=167).

(E) 3D reconstruction images of the representative clones with an anti-correlation between adjacent layers (Left: L6 versus L5; Middle: L5 versus L4; Right: L4 versus L2/3). Colored lines indicate the layer boundary and colored dots represent the cell bodies of labeled neurons. Neurons in the adjacent layers are circled with white broken lines.

(F) Heatmap of the correlation between neuron numbers in different layers in E12 clones (n=167). Colors represent the correlation coefficiency based on Pearson's correlation test. Corresponding p values are shown in the heatmap. Note that only the adjacent layers (L6 versus L5, L5 versus L4, L4 versus L2/3) exhibit a significant anti-correlation.

(G) Pearson's correlation between all adjacent (n=501) and non-adjacent (n=501) layer pairs in E12 clones. Bar plots represent the correlation coefficiency.

(H) Heatmap of the correlation between neuron numbers in different layers in a random simulated dataset (n=167 for 100 trails). Colors represent the correlation coefficiency based on Pearson's correlation test. Corresponding p values are shown in the heatmap.

(I) Pearson's correlation between all adjacent (n=501 for 100 trails) and non-adjacent (n=501 for 100 trails) layer pairs in a random simulated dataset. Bar plots represent the correlation coefficiency.

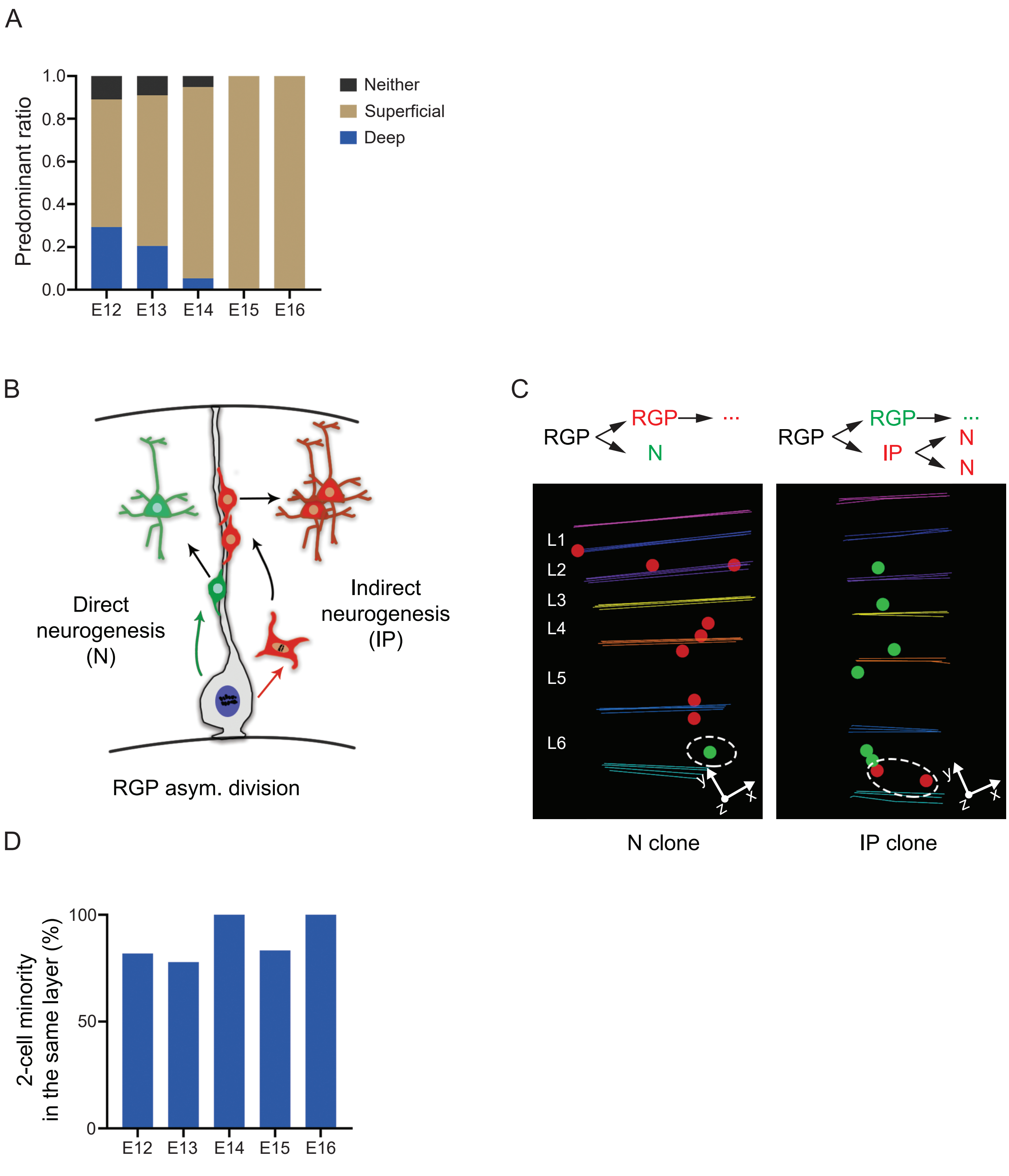

Shen et al., Figure S4

Figure S4. Direct and indirect neurogenesis by RGPs.

(A) Ratios of the clones with the predominant neuronal output to the superficial or deep layers labeled at different developmental stages (E12, n=167; E13, n=131; E14, n=33; E15, n=44; E16, n=31).

(B) Schematic diagram of the asymmetric division of RGPs with direct or indirect neurogenesis. N, neuron; IP, intermediate progenitor; RGP, radial glial progenitor.

(C) 3D reconstruction images of the representative clones with direct (left) or indirect (right) neurogenesis at the 1st division. Colored lines indicate the layer boundary and colored dots represent the cell bodies of labeled neurons. The minority color labeled neurons are circled by white broken lines. Schematics of the clones are shown at the top. RGP, radial glial progenitor; N, neuron; IP, intermediate progenitor.

(D) Percentages of the clones with the minority color labeled neurons located in the same layer at different developmental stages.

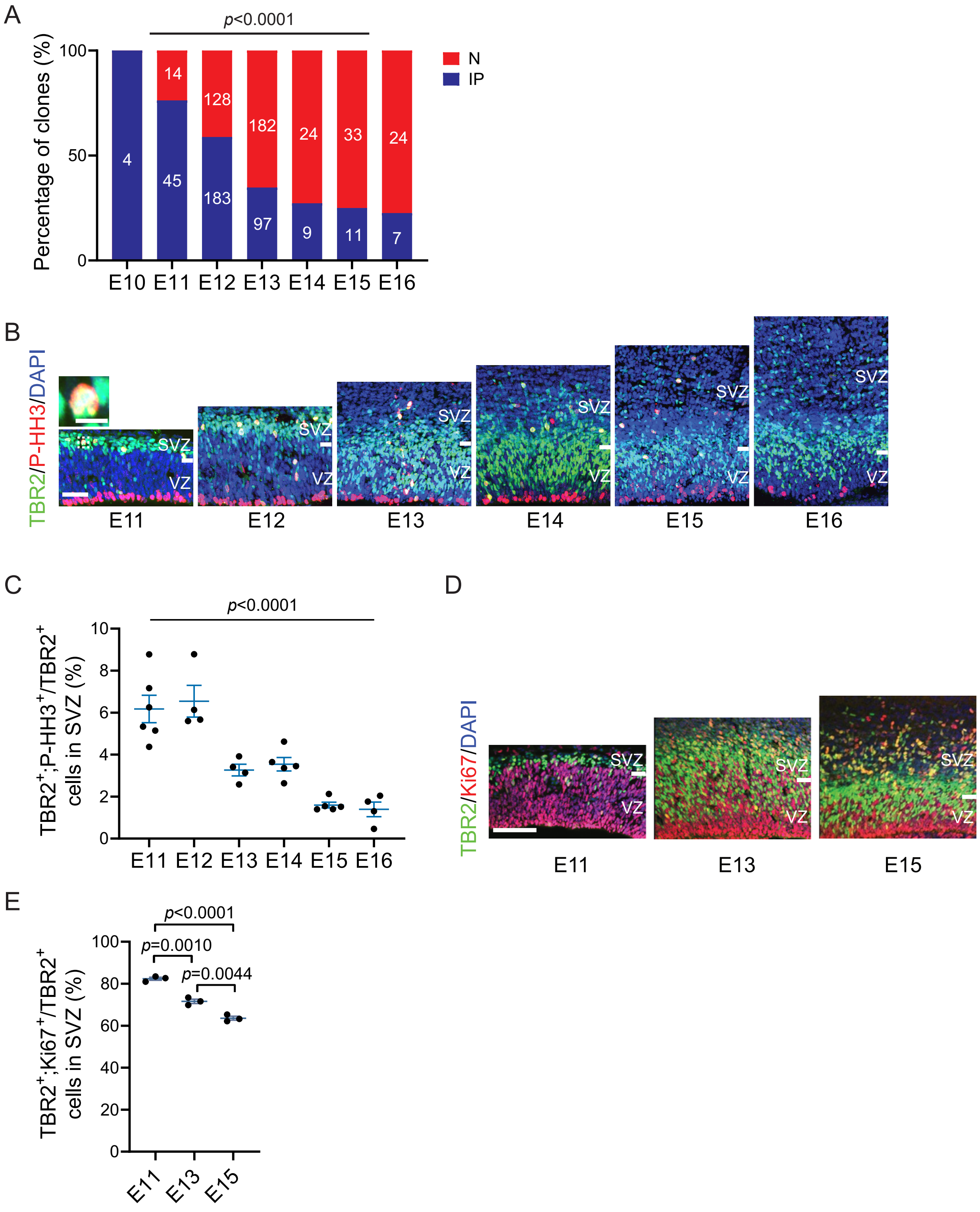

Shen et al., Figure S5

Figure S5. Progressive change of ratio of direct and indirect neurogenesis.

(A) Ratios of direct neurogenesis (N) and indirect neurogenesis (IP) indicated by the minority color labeling neuronal number in individual MADM clones labeled at different developmental stages (E10, n=4; E11, n=59; E12, n=311; E13, n=279; E14, n=33; E15, n=44; E16, n=31). Statistical analysis was performed with Chi-square.

(B) Representative confocal images of the VZ/SVZ of the developing neocortices at different developmental stages stained for TBR2 (green), P-HH3 (red), and DAPI (blue). A high-magnification image (dashed line square) of a representative $TBR2^+$;$P\text{-}HH3^+$ cell is shown at the top. Scale bars: 10 μm (top) and 50 μm (bottom).

(C) Quantifications of the percentages of $TBR2^+$;$P\text{-}HH3^+$ cells out of total $TBR2^+$ cells in SVZ (E11, n=6; E12, n=4; E13, n=4; E14, n=5; E15, n=5; E16, n=4). Lines and error bars represent mean ± SEM. Statistical analysis was performed with one-way ANOVA.

(D) Representative confocal images of the neocortices at E11, E13, and E15. Sections were stained for TBR2 (green), Ki67 (red), and DAPI (blue). Scale bar: 100 μm.

(E) Quantifications of the percentages of $TBR2^+$;$Ki67^+$ cells of the total $TBR2^+$ cells in the SVZ (E11, n=3; E13, n=3; E15, n=3). Bar plot and error bars represent mean ± SEM. Statistical analysis was performed with one-way ANOVA.

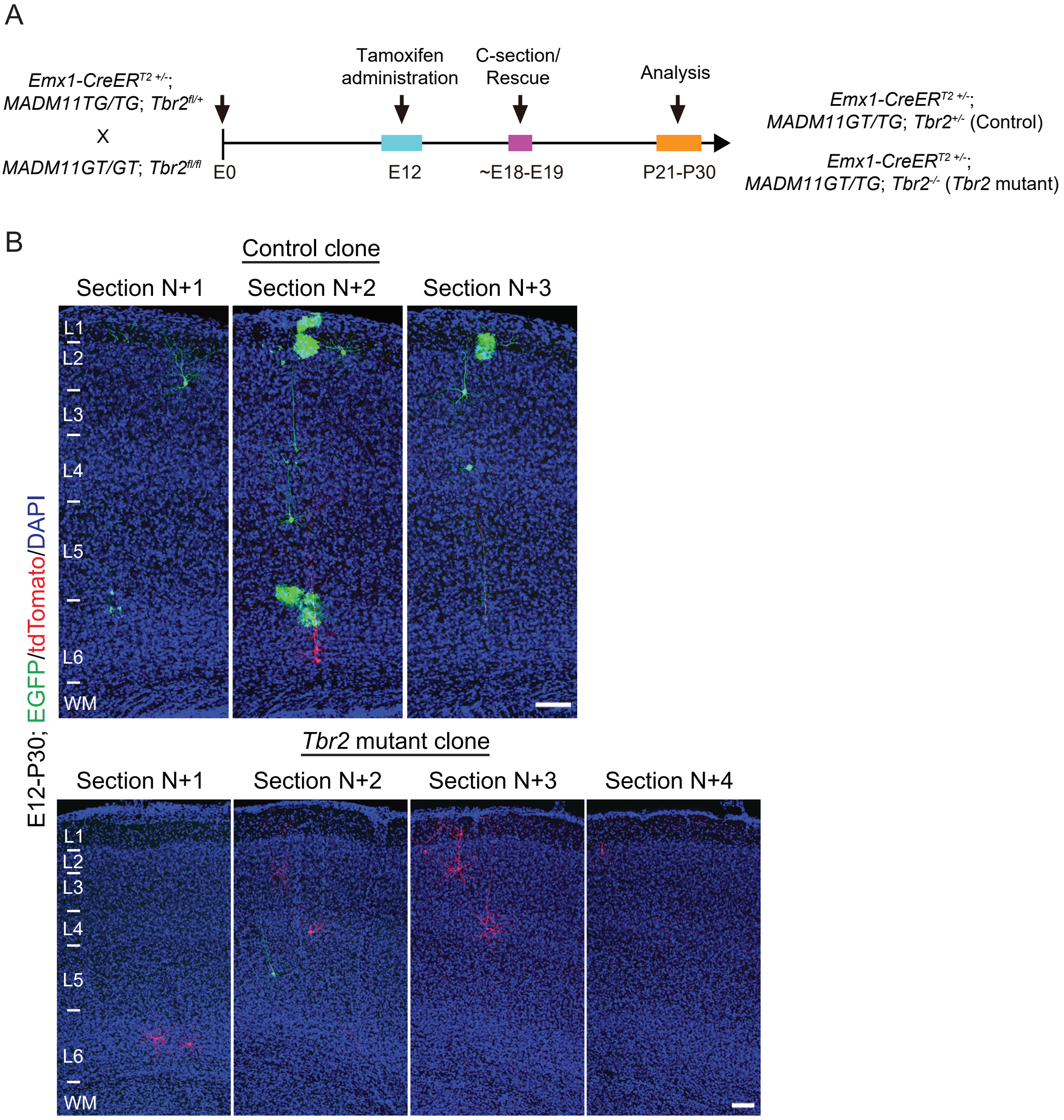

Shen et al., Figure S6

Figure S6. MADM labeling of control and *Tbr2* mutant RGP clones in the neocortex.

(A) Experimental diagram of MADM-based clonal analysis in the control and *Tbr2* Mutant neocortices.

(B) Representative confocal images of the P30 control (top) and *Tbr2* mutant (bottom) clones labeled at E12. Consecutive sections were stained for EGFP (green) and tdTomato (red), and counterstained with DAPI (blue). L, layer; WM, white matter. Scale bar: 200 μm.

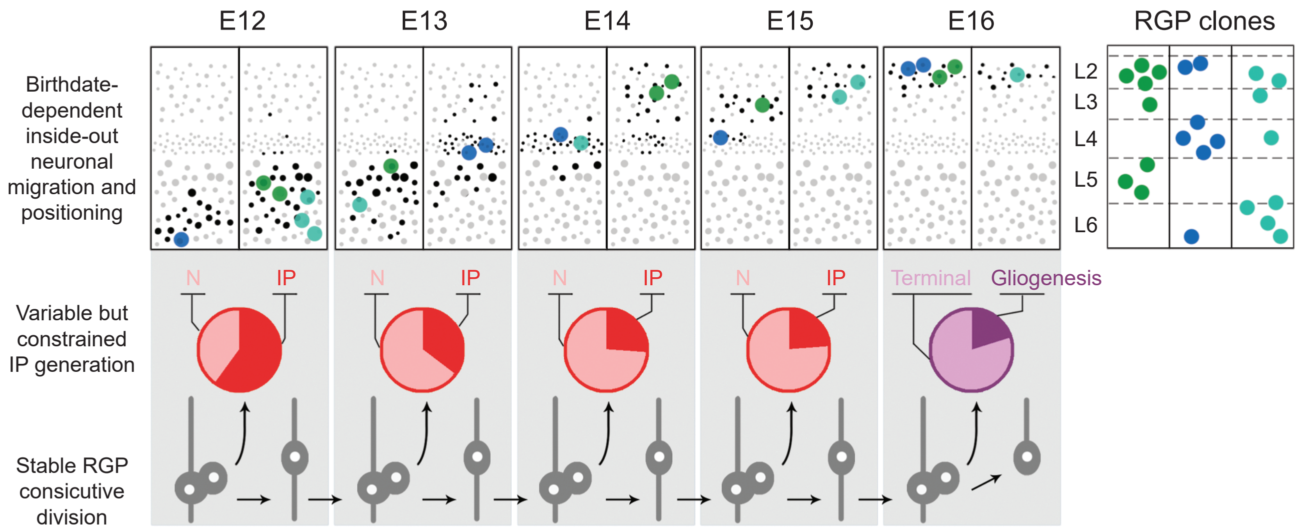

Shen et al., Figure S7

Figure S7. Summary of the basic neurogenesis features for a balanced reliability and robustness in the assembly of the complex neocortex.

A stable consecutive RGP asymmetric division framework, which supports for the reliability of neurogenesis, and a variable but constrained IP generation, which accounts largely for the variability and robustness of neurogenesis in the formation of all layers, in conjunction with the birthdate-dependent inside-out migration and positioning of newborn neurons support the effective assembly of the complex neocortex. During the process, individual RGPs give rise to clones with constrained and patterned variabilities in neuronal number and layer composition (inset).
